# Large Electrocaloric Responsivity and Energy Storage Response in the Lead-Free Ba(Ge*_x_*Ti_1−*x*_)O_3_ Ceramics

**DOI:** 10.3390/ma15155227

**Published:** 2022-07-28

**Authors:** Bouchra Asbani, Yaovi Gagou, Said Ben Moumen, Jean-Luc Dellis, Abdelilah Lahmar, M’Barek Amjoud, Daoud Mezzane, Mimoun El Marssi, Brigita Rozic, Zdravko Kutnjak

**Affiliations:** 1 Laboratoire de Physique de La Matière Condensée (LPMC), Université de Picardie, Jules Verne, 33 rue Saint-Leu, CEDEX 1, 80039 Amiens, France; asbani.bouchra@gmail.com (B.A.); dellisjeanluc@gmail.com (J.-L.D.); lahmar2@yahoo.fr (A.L.); mimoun.elmarssi@u-picardie.fr (M.E.M.); 2 Unit of Dynamics and Structure of Molecular Materials—UDSMM (EA 4476), MREI-1, Université du Littoral Côte d’Opale, 59140 Dunkerque, France; 3 IMED-Lab, Department of Applied Physics, Faculty of Sciences and Techniques, Cadi Ayyad University, P.O. Box 549, Marrakesh 40000, Morocco; said.benmoumen@yahoo.fr (S.B.M.); m.amjoud@uca.ac.ma (M.A.); daoudmezzane@gmail.com (D.M.); 4 Jozef Stefan Institute, Jamova Cesta 39, 1000 Ljubljana, Slovenia; brigita.rozic@ijs.si (B.R.); zdravko.kutnjak@ijs.si (Z.K.)

**Keywords:** BGT, ceramics, ferroelectric, electrocaloric, energy storage

## Abstract

Ferroelectric property that induces electrocaloric effect was investigated in Ba(Ge*_x_*Ti_1−*x*_)O_3_ ceramics, known as BTGx. X-ray diffraction analysis shows pure perovskite phases in tetragonal symmetry compatible with the *P*4*mm* (No. 99) space group. Dielectric permittivity exhibits first-order ferroelectric-paraelectric phase transition, confirmed by specific heat measurements, similar to that observed in BaTiO_3_ (BTO) crystal. Curie temperature varies weakly as a function of Ge-content. Using the direct and indirect method, we confirmed that the adiabatic temperature change Δ*T* reached its higher value of 0.9 K under 8 kV/cm for the composition BTG6, corresponding to an electrocaloric responsivity Δ*T/*Δ*E* of 1.13 × 10^−6^ K.m/V. Such electrocaloric responsivity significantly exceeds those obtained so far in other barium titanate-based lead-free electrocaloric ceramic materials. Energy storage investigations show promising results: stored energy density of ~17 mJ/cm^3^ and an energy efficiency of ~88% in the composition BTG5. These results classify the studied materials as candidates for cooling devices and energy storage applications.

## 1. Introduction

Electrocaloric effect (ECE) is similar to magnetocaloric effect (MCE), in which the temperature change of the material is achieved by the electrical and magnetic field control, respectively [1,2,3]. Therefore, ECE occurs in switchable dipolar materials, where the application of electric field leads to change in the reversible polarization and consequently in the entropy [4]. Briefly, the applied electric bias induces an adiabatic increase of temperature in the ferroelectric material. The field removal causes similarly adiabatic cooling in a reversible process known as ECE [5,6,7].

Nowadays, ECE attracts scientists’ attention because of its promising frigorific applications and microelectronic cooling devices [8,9,10]. Various contributions were recently reported on ferroelectric thin films, ceramic and polymers [1,7,11,12].

Prototype refrigerators using ceramic ECE have been experimented with by a few research groups [13,14,15]. The low ECE in these materials prevent them from being used in efficient calorimetric applications. Therefore, high performance solid-state cooling devices could be realized only using materials with large ECE, for example, in electronic elements cooling of microelectronic devices, sensors, textile, etc.

Moreover, electrocaloric (EC) coolers based on eco-friendly materials stimulate a resurgent focus on the development of high-performance lead-free barium titanate BaTiO_3_ (BTO)-based materials [12,13,14,15] due to the cancerous nature and neurotoxicity of Lead (Pb) [16,17].

BTO is one of the most frequently used ceramic materials in electronic devices due to its outstanding dielectric properties [18]. In particular, remarkable tailoring of functional characteristics can be achieved by substitution with foreign ions on the A, B, or in both sites of the ABO_3_ perovskite structure. Isovalent substitution ions such as Zr^4+^, Sn^4+^ and Ce^4+^ onto the Ti^4+^ site gave rise to enhanced material properties (e.g., dielectric, pyro- and piezoelectric), high thermal stability in a large temperature range [2,4]. Otherwise, substitution can induce also a decrease in polarisation [2], tunability [19] and hysteresis loops [20]. These substitutions can also induce structural-phase transitions or polymorphic phases overlapping for specific compositions, and sometimes a ferroelectric relaxor by increasing the dopant concentration [21]. Substitutions with smaller radius ions such as Ge are far less investigated and apparently do not modify the structural transition temperatures: the room temperature tetragonality and the ferroelectric character [22]. There are only a few reports concerning the role of Ge insertion, which has mainly been used as sintering aids to promote better densification of BTO-based ceramics at a lower sintering temperature [23]. Chu et al. [24] reported that germanates are used in industrial applications to produce heterophasic ceramic bodies. Köferstein et al. [25] have investigated the influence of BaGeO_3_ (BGO) on the sintering behaviour and properties of fine- and coarse-grained BTO powder density. GeO_2_ can be used as a sintering aid to reduce the sintering temperature of BTO ceramics below 1000 °C. Guha and Kolar [26] studied the BaTiO_3_−BaGeO_3_ (BTO-BGO) system, and they determined a eutectic composition of 68 mol% BaGeO_3_ with a melting temperature of approximately 1120 ± 5 °C. The authors did not observe any shifting of the cubic to the tetragonal phase transition temperature in BTO by the addition of GeO_2_, in agreement with the investigations by Plessner and West [27]. These authors noticed a reduction in the sharpness of the permittivity maximum. In contrast, Pulvari [28] and Baxter [29] found a slight decrease in the Curie temperature with the addition of GeO_2_, and determined the limit of solubility of some doping elements in BTO. Consequently, the knowledge about the effect of sintering additive materials on the dielectric properties of BTO-based ceramics is essential for potential technological applications.

Therefore, ECE is evidenced in BTO-doped ferroelectric material using indirect or direct methods [30]. The indirect method used in our case is based on thermal P-E hysteresis loop data analysis using Maxwell relations [31]. In addition, the results were confirmed by direct method using a modified high-resolution calorimeter [32].

The literature indicated that the EC responsivity Δ*T*/Δ*E* is the most suitable coefficient to highlight the electrocaloric effect [33,34,35]. This is supported by the aim of producing high ECE under optimal applied electric field. The electrocaloric effect in Na_0.5_Bi_0.5_TiO_3_–BaTiO_3_ (NBT-BTO) [30] and BTO bulk ceramics had recently been investigated by Bai et al. [7], who reported a maximum electrocaloric responsivity of 8 × 10^−7^ K.m/V. Wang et al. [36] obtained a Δ*T*/Δ*E* value of 1.5 × 10^−7^ K.m/V in the Ba_0.98_Ca_0.02_(Zr_0.085_Ti_0.915_)O_3_ (BCZT) ceramics. Liu et al. [37] reported that ECE responsivity in Ba_0.65_Sr_0.35_TiO_3_ (SBT) ceramics prepared by spark plasma sintering was enhanced up to 2.33 × 10^−7^ K.m/V. In BTO single crystal, Novak et al. [6] evidenced a Δ*T*/Δ*E* value of 5 × 10^−7^ K.m/V by high-resolution EC measurements in the narrowed region of transition. We reported previously a large ECE in a much broader temperature range extended in 50 K a maximum Δ*T*/Δ*E* of 3.4 × 10^−7^ K.m/V in Ba_0.8_Ca_0.2_TiO_3_ Zr-doped, lead-free ferroelectric ceramics at a relatively small electric field of 7.95 kV/cm [34]. Previous results demonstrate a significant challenge to achieving high EC adiabatic temperature changes in the lead-free bulk materials with the optimal applied electric field and the best doping cation for higher EC responsivity. It is worth noting that the optimal electric field minimises material fatigue and Joule heating effect. Operating at low voltage could also be a suitable way to reduce the technology cost and ensure low energy consumption.

In the present paper, we focus our investigation on the ECE and energy storage performance in BTGx materials (*x* = 0.02, 0.03, 0.05, 0.06 and 0.09). We study the influence of germanium (Ge) doping in BT matrix on the structural, electrical, ferroelectric, electrocaloric and stored energy properties in this system. It should be noted that ECE results obtained using indirect methods were found to be in good agreement with data directly measured using a high-resolution calorimeter, and the energy stored shows promising results.

## 2. Experimental Section

BTGx ceramics were elaborated using a conventional powder processing technique and starting from high purity raw materials BaCO_3_ (99%), TiO_2_ (99.8%) and GeO_2_ (99.8%), which were mixed in the desired stoichiometry. The mixture powder was grounded in ethanol medium for an hour in agate mortar, followed by a thermal treatment. The obtained powder of each Ge-content was calcined at 900 °C for 4 h. Heating rate was 5 °C/min. This thermal process had been repeated three times for grain size reduction and for accurate grain reactions to achieve a homogenous state with single-phase powder.

The symmetry phases were confirmed by X-ray powder diffraction (XRD) data analysis which was recorded with detector step of 0.0199° and waiting time of 10 s using Brucker D8 with λ_Cu_ = 1.5406 Å in *θ-2θ* Bragg–Bretano geometry configuration. The structural resolution was carried out using the Rietveld method implemented in the FullProf software [38]. The finely granulated powder was compacted under a hydraulic press at 250 MPa pressure to obtain circular pellet discs of 6 mm diameter and 0.4 mm thickness. The obtained pellets were then placed into an alumina crucible and sintered at 1100 °C for 1 h. The obtained sintered ceramic samples were crack-free, and their density was found in the range 5.03 to 5.80 g/cm^3^. These densities measured based on Archimedes’ method, to represent 93 to 96% of their theoretical value. Silver paste was used to form electrodes covering both faces of the pellets to form a plane capacitor shape for electrical measurements. Measurements can be started after 30 min heating of electrodes at 300 °C constant temperature. Then, P-E hysteresis loops were registered as a function of temperature using AixACCT TF1000 apparatus, and dielectric permittivity was measured for several frequencies (100 Hz–1 MHz) as a function of temperature using Solartron SI 1200 Impedancemeter. Temperature controller is Linkam THMS600 with heating and cooling rate of 5 °C/min. Temperature module offered 0.01 °C in accuracy. All the thermal measurements have been performed under air. The Impedance measurements were performed every 2.00 °C ± 0.01 °C, under oscillator source of 50 mV applied on ~0.5 mm sample thickness. Sample heat capacity was deduced from heat flow measurements performed using Netzsch DSC 204F1 apparatus. SEM images were recorded under 5 kV electrons acceleration source and a working distance in the range 10–15 mm. The Energy Dispersive X-ray (EDX) analysis had been performed using the FEI Quanta 200F apparatus.

## 3. Structural Studies

### 3.1. X-ray Studies

**Figure 1:**
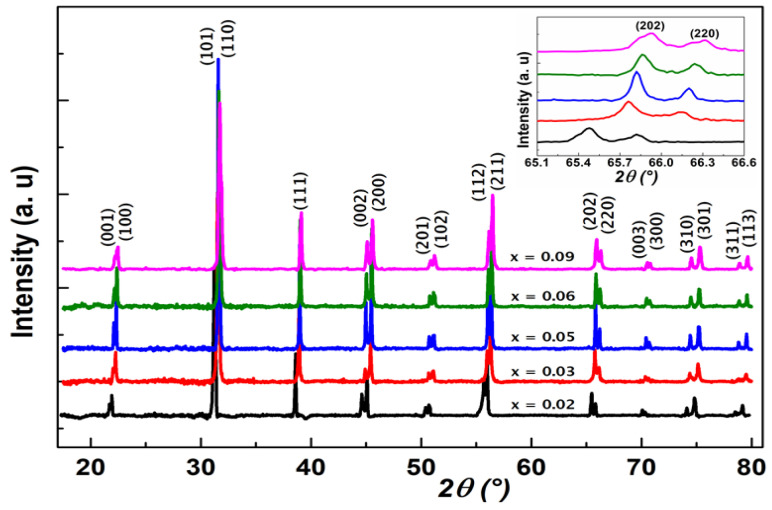
Room temperature X-ray patterns of elaborated compound BTGx (x = 0.02, 0.03, 0.05, 0.06, 0.09). The inset indicates the zoom of evolution of (202) and (220) lines versus Ge-content.

X-ray diffraction patterns of BTGx ceramics (x = 0.02, 0.03, 0.05, 0.06, 0.09) performed at room temperature were shown in Figure 1. Small displacement of the rays to high angles can be observed in this figure versus Ge-content, which results in the contraction of lattice parameters. No significant structural change was observed at first look based on the diffraction lines. This means Ge-insertion in BTO matrix induces weak distortion in the crystal lattice parameters, and the tetragonal symmetry was observed for all the compositions. A zoom conducted on the tetragonal symmetry lines (202) and (220) showed a weak displacement of ~0.5°, as presented in the inset of Figure 1. The structural resolution was then conducted for all the elaborated compositions satisfactorily. Beginning from profiles adjustment, the calculations led to rapid convergence. By way of the Rietveld method using FullProf software, the atomic positions, thermal isotropic agitation factors, occupation rates and scale factor were adjusted at room temperature to minimize the reliability factors in a coherent way, based on BTO tetragonal matrix in accordance with the *P*4*mm* (No. 99) space group and JCPDS N° 05-0626. We observed globally a small decrease of lattice parameters leading to a small decrease in lattice volume. This behaviour can be expected, since the ionic radius [39] of Ge^4+^ (0.530 Å) in substitution of Ti^4+^ ion in the octahedral site is slightly smaller than Ti^4+^ ionic radius (0.605 Å), which could lead to a decrease of volume if the substitution occurred. It is worth recalling that a systematic microstructural analysis and the phase equilibrium diagram for the system BaTiO_3_-BaGeO_3_ (BTO-BGO) was reported by Guha et al. [26], in which the solubility limit of BGO in BTO was determined around 1.8 mol% but no electrical characterization was performed. In the present case, structural resolution leads to several observations: (i) small octahedra distortion is observed, (ii) volume decreases weakly, (iii) Ge doping atoms in the octahedral site have weak concentration and consequently do not impact greatly the structural symmetry.

**Figure 2:**
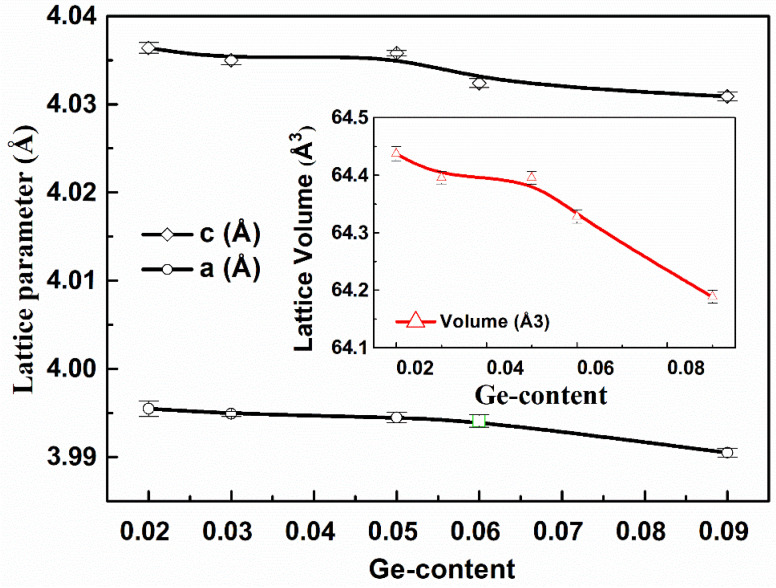
Lattice parameters (a and c) and volume variation of BTGx (x = 0.02, 0.03, 0.05, 0.06, 0.09) versus Ge-content. These parameters were obtained from Rietveld structural resolution using FullProf software.

The refined lattice parameters (a = b and c), the unit cell volume, atomic positions and the reliability factors are summarized in Table 1, and the curves of evolution of lattice parameter and volume versus Ge-content are presented in Figure 2. Moreover, considering the ionic radius of chemical elements and Ge-content, we calculated the Goldschmidt tolerance factor to characterize the perovskite structural stability. The obtained values gathered in Table 1, demonstrate progressive stabilization to pseudo-cubic perovskite structure while Ge-content increases.

### 3.2. Raman Spectroscopy

**Figure 3:**
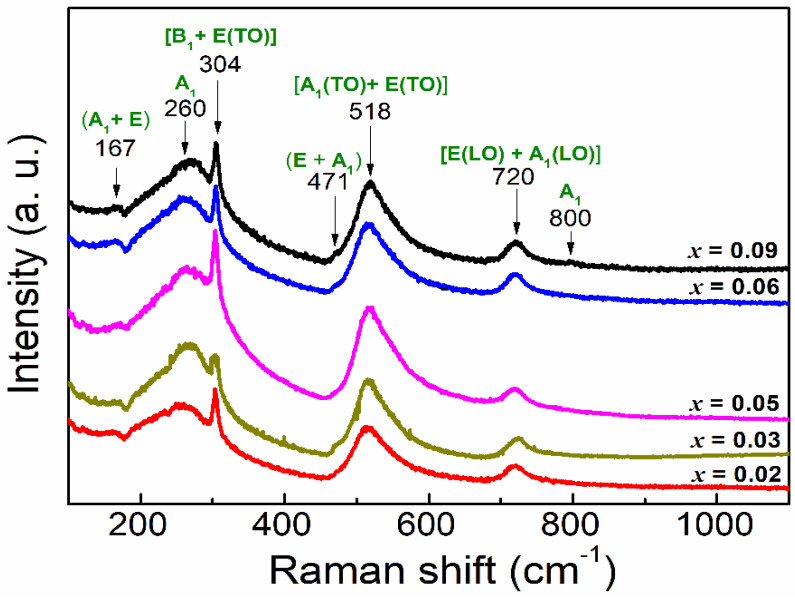
Room temperature Raman spectra for the studied BTGx (x = 0.02, 0.03, 0.05, 0.06, 0.09) compositions. The A_1_(TO) mode at 800 cm^−1^ confirms Ge-insertion in the octahedral site in the perovskite.

To confirm the structural analysis, we performed Raman spectroscopy measurements at room temperature. The spectra are plotted in Figure 3 and show the BTO known active modes in its tetragonal symmetry (C_4v_) for all the compositions as reported in the literature [40,41,42,43]. The active modes are covered in the frequency range 100–800 cm^−1^ [41,44]. Three E(TO) modes appear around 167, 304 and 518 cm^−1^, which were often observed at 170, 306 and 520 cm^−1^ in pure BTO ceramics [44]. Moreover, the modes 260, 471 and the somewhat broader 720 cm^−1^ constitute the A_1_ modes in this structure. An extra A_1_ mode appears clearly at 800 cm^−1^ for composition x = 0.09. This mode is sensitive to Ge-rate, and very close observation indicates that it occurs earlier at lower concentration with weak intensity and evolves to be observable at x = 0.09. It was previously reported [45] that this mode is sensitive only to B-site occupation in perovskite matrix and move to high frequency as a function of BTO-doping rate and was affected to the A1(TO) mode. This confirms that Ge is inserted in the B-site of the perovskite structure [45]. Therefore, we can conclude that no significant displacement or variation of the modes is observed with Ge-content until x = 0.09, where the A1(TO) mode appears. This can be explained by the weak Ge-doping, which corroborates the X-ray analysis.

### 3.3. Microstructure Analysis

**Figure 4:**
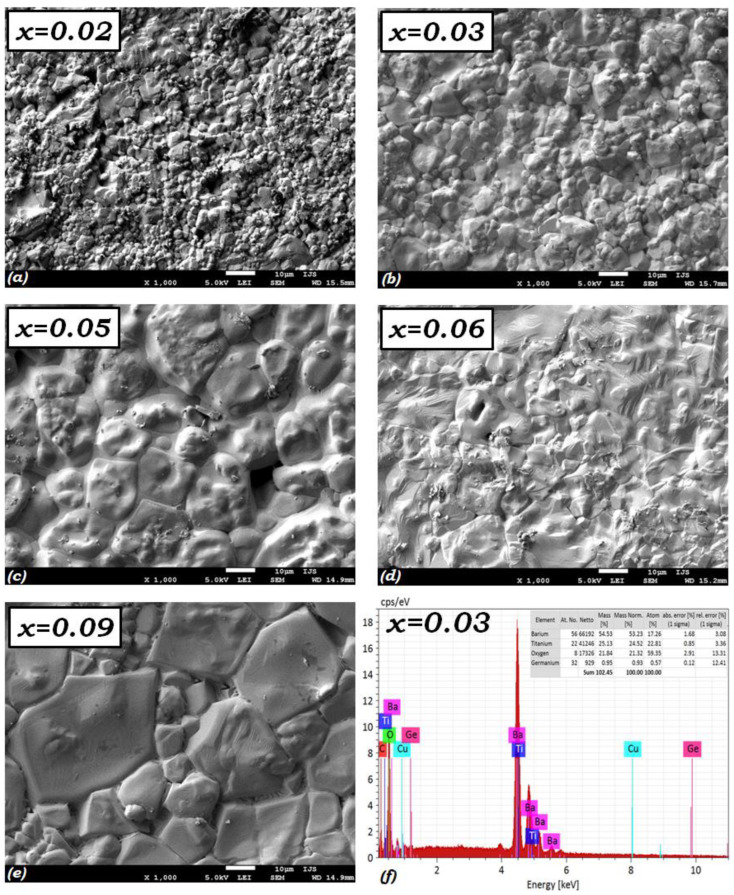
(**a**–**e**) SEM micrographs of BTGx (x = 0.02, 0.03, 0.05, 0.06, 0.09) ceramics and (**f**) EDX analysis of the composition BTG3 showing mass percentage of containing elements in agreement with the theoretical values.

We present in Figure 4 the SEM micrographs of the five elaborated BTGx (x = 0.02, 0.03, 0.05, 0.06, 0.09) ceramics. These images show a significant variation in grain size depending on the composition. We can observe the increase of grain size with Ge-content, except from the particular grain size obtained for the composition BTG6. The grains seem to bathe in a lacquer, assuming the relative compacity of the structure with Ge-content. This behaviour can explain the effect of Ge-content around the limit of solubility of BGO in BTO matrix. The grain bonding defects disappear at the interfaces, leading to acceptable density of the ceramics with Ge-content. Moreover, the density of the ceramics was calculated based on the Archimedes method, and the values range between 93–96% of their theoretical values, as reported in Table 1. A typical morphology was observed in the BTG6 ceramic, which also exhibits particular behaviour in X-ray analysis and other results. Chemical analysis based on Energy Dispersive X-Ray (EDX) analysis confirmed the compositions, as we can observe at bottom right part of Figure 4 for the composition BTG3. Experimental relative weight values are close to theoretical ones, which are: 58, 19, 20, and 0.9% mass percentage, for the chemical element Ba, Ti, O and Ge, respectively (see inset of Figure 4f). Furthermore, the density values range in 5.035 to 5.801 g/cm^3^ and the grain sizes determined using ImageJ software range between 3.6 to 18.7 µm. The values for each composition are depicted in Table 1.

## 4. Dielectric Measurements

**Figure 5:**
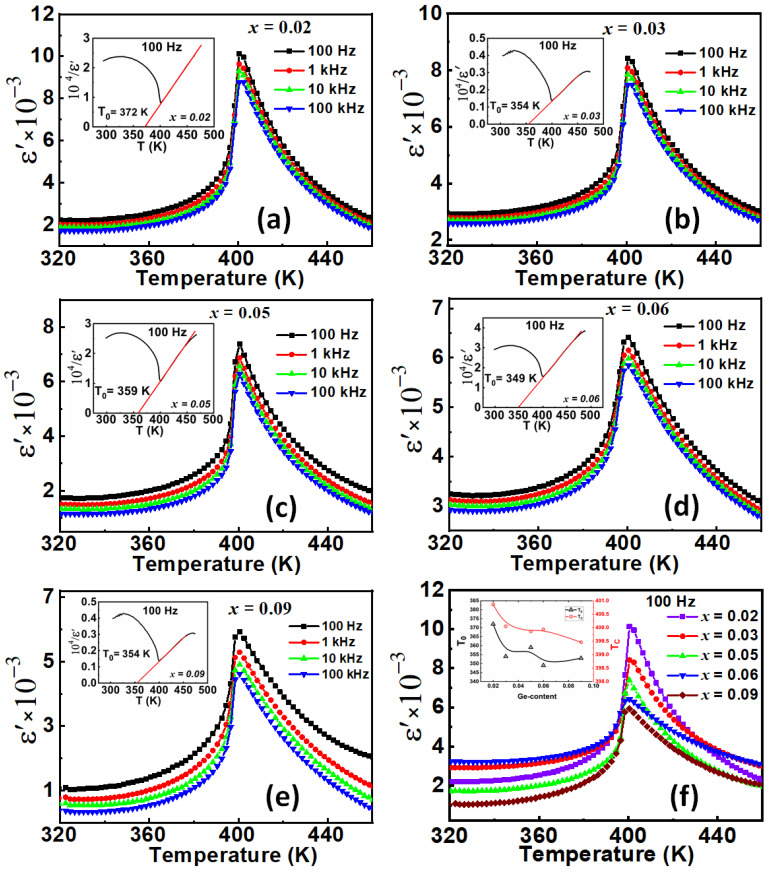
(**a**–**e**) Temperature dependence of dielectric permittivity in BGTx (x = 0.02, 0.03, 0.05, 0.06, 0.09) ceramics in the frequency range 10^2^–10^5^ Hz. Insets represent the Curie–Weiss plot of each composition. (**f**) Dielectric permittivity at 100 Hz for all the BTGx compositions. The inset shows T_C_ and T_0_ variation as a function of Ge-content.

Figure 5 displays the temperature dependence of the dielectric permittivity measured at different frequencies for all the ceramics. The Ferroelectric-to-Paraelectric (FE-PE) structural phase transition is marked by the dielectric permittivity jump at Curie temperature (T_C_). The symmetry changes from tetragonal *P*4*mm* (No. 99) to cubic *Pm*-3*m* (No. 221) space groups in the paraelectric phase. The real part of relative dielectric permittivity of the BGTx ceramics reaches at T_C_ a high value of 10,140 for the composition BTG2 and then decreases to 5918 for BTG9, with a very weak variation of the Curie temperature between the studied compounds. Moreover, no relaxor effect was observed in this system, which can be considered as classical ferroelectric material. Horchidan et al. [21] reported that for small Ge additions to BTO x ≤ 0.10, for which the perovskite tetragonal phase is predominant, the dielectric properties are quite similar to ones of BTO ceramics, with all the structural phase transitions in the same temperature range and a small shift of the Curie temperature to higher with Ge-content. This seems to be in contradiction with the present results. T_C_ decreases weakly when Ge-content increases. Furthermore, the structural phase transition is of first-order type, as evidenced by the drastic dielectric permittivity jump and the thermal hysteresis observed for all the compositions. Curie temperature remains almost constant in-between temperature variation ΔT_C_ = 1 K for these studied Ge-contents. Plessner et al. [27] reported a similar result on electrical measurement, showing no significant variation of Curie temperature with Ge-rate. We confirm this result by the specific heat variation depicted from DSC Signal measurements that evidenced, for all the compositions, an asymmetric peak versus temperature, in favour of first order type phase transition as shown later in this work. Although the Curie temperature varies almost weakly, for all the samples we observed variation of the Curie constant, reflecting the mechanism of the different dynamics of phase transitions in these samples, especially in BTG2 and BTG9. The Curie–Weiss temperature T_0_ in this system presents its low value for the composition BTG6, as indicated in Table 1.

We also report in Table 1 the Curie constant values and the Curie temperatures obtained from dielectric permittivity measurements. The higher gap value (~51 K) between T_C_ and T_0_ is observed for the composition BTG6. This composition constitutes that in which metastable transformation exists in a large range temperature, one of the reasons for the better electrocaloric adiabatic temperature variation, but not beneficial to energy storage.

**Figure 6:**
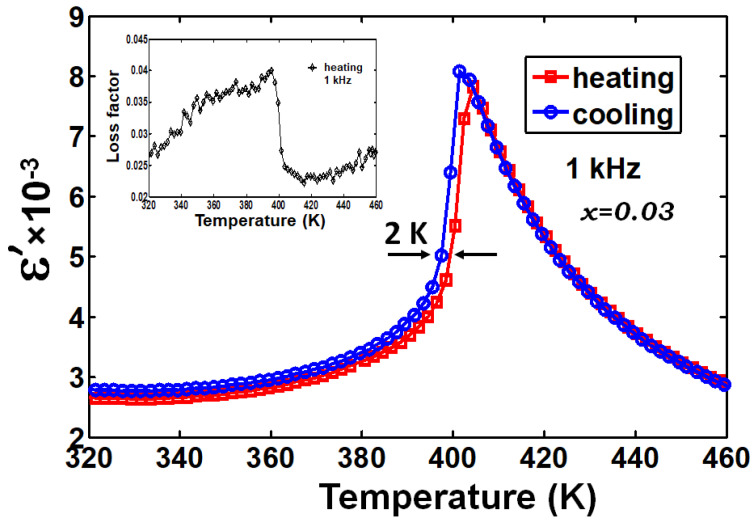
Thermal hysteresis loop of real permittivity for BTG3 compound, recorded at 1 kHz, showing a discard of ΔT_C_ = 2.00 K between heat and cool data. Inset the dielectric shows Loss factor.

Samples globally presented a real permittivity thermal hysteresis of about 2 K, as showed in Figure 6 for the composition BTG3 recorded at 1 kHz. The inset of this figure also highlights a global weak loss factor less than 4.1%.

## 5. Ferroelectricity and Electrocaloric Effect

### 5.1. Ferroelectric Properties

**Figure 7:**
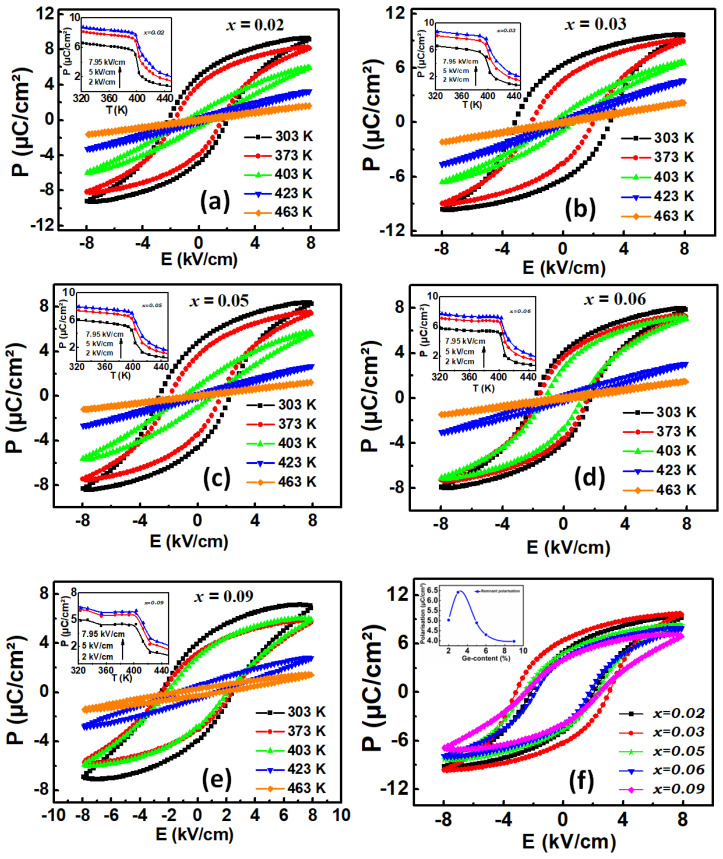
(**a**–**e**) P-E hysteresis loops of BTGx ceramics (x = 0.02, 0.03, 0.05, 0.06, 0.09) as a function of temperature and extracted maximum polarization (insets) variation versus temperature at three applied fields. (**f**) Comparative hysteresis loops at T = 303 K and remnant polarization (inset) versus Ge-content.

P-E hysteresis loops were recorded on cooling in the temperature range from 473 to 273 K to minimize the polarization inaccuracy induced by fatigue during heating. Figure 7 presents the P-E hysteresis loops variation recorded at 5 Hz in the BTGx samples as a function of temperature. These curves show a ferroelectric character for T < T_C_, confirmed by the non-linear behaviour, and then evolves towards a paraelectric phase for T > T_C_, characterized by the linear curve. In the insets of Figure 7, we present the polarization variation versus temperature under three selected applied electric fields. We remark on the decrease of polarization as a function of temperature followed by an abrupt drop at Tc, for all the compositions (Figure 7a–e), which was particularly important for the compound BTG6. A particularly rapid jump was observed at T_C_ for the composition BTG6, and therefore, the highest value of the pyroelectric coefficient *dP/dT*, favouring a significant electrocaloric effect, was observed for this composition.

In Figure 7f, we present comparative P-E hysteresis loops for all the compositions at room temperature (T = 303 K). The lowest coercive field Ec value is observed for BTG6. The inset of Figure 7f shows the evolution of remnant polarization versus Ge-content that exhibits a maximum at the composition BTG3, before decreasing to a low value when Ge-content increases. This behaviour is attributed to dipole reordering and domain wall motion mechanism versus Ge-content.

### 5.2. Indirect Electrocaloric Effect

**Figure 8:**
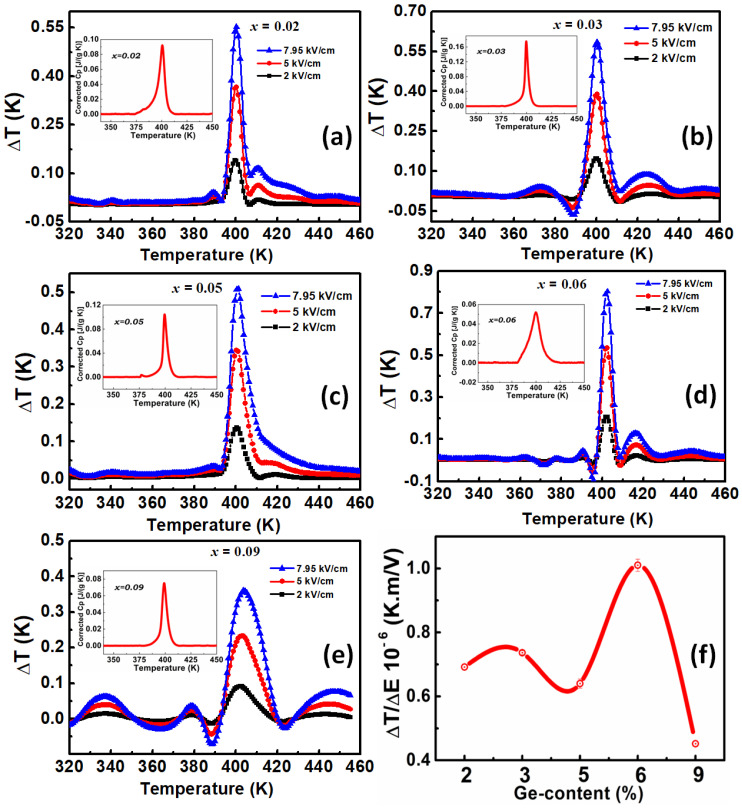
(**a**–**e**) Electrocaloric adiabatic temperature change (ΔT) as a function of temperature at three different applied electric fields (2.00, 5.00 and 7.95 kV/cm). The insets show corrected heat capacity Cp deduced from heat flow measurement versus temperature. (**f**) Electrocaloric responsivity as a function of Ge-content.

The electrocaloric effect was then investigated by an indirect method deduced from P-E hysteresis measurements. We extracted the upper branches from the temperature-dependent P-E hysteresis loops to calculate the variation of polarization versus temperature *P*(*T*). The pyroelectric coefficient *∂P/∂T* is then calculated from fourth-order polynomial fits of the *P*(*T*) data, and the adiabatic electrocaloric temperature change (Δ*T*) was deduced from this analysis according to the equation:

(1) $$\Delta T=-\frac{1}{\rho }\overset{E_{2}}{\underset{E_{1}}{\int }}\frac{T}{c_{p}}{\left(\frac{\partial P}{\partial T}\right)}_{E}dE$$

where ρ is the density of each studied material, E_1_ and E_2_ are the starting and the final applied electric fields, respectively, and *Cp* is the specific heat capacity of each studied material. Figure 8a–e show the electrocaloric adiabatic temperature change as a function of temperature for all studied Ge-doped compounds at three selected applied electric fields: 2.00, 5.00 and 7.95 kV/cm. The absolute maximum of each obtained ∆*T* curve occurs at FE-PE phase transition temperature. The insets show heat capacity *Cp* deduced from heat flow measurement versus temperature, which were adjusted to their background polynomial fits. Note that electrocaloric effect depends mainly on excess of specific heat at phase transition. The higher value of ∆*T* was evidenced for the composition BTG6, which reached the value of 0.8 K. This high value is expected at this particular composition, since a drastic drop was observed in the polarisation at T_C_ and also due to its particular crystallinity. A broad anomaly in ∆*T* was observed for BTG9, as shown in Figure 8e, attributed to the limit of Ge substituting in the BTO matrix that induced conductivity which appears in the less saturated P-E hysteresis or the broad specific heat curve in the inset of Figure 8e. The evolution of maximal variation of electrocaloric responsivity as a function of Ge-content is plotted on Figure 8f.

In the BGTx system, the calculation of electrocaloric responsivity leads to a high value of ΔT/ΔE=1.01 K.m/V at 400 K in BTG6. This electrocaloric responsivity was obtained just under an applied electric field value of 7.95 kV/cm. To our knowledge, this value is one of the higher values of electrocaloric responsivity reported in lead-free barium-based oxides, making the BTGx system a candidate for refrigeration devices.

### 5.3. Direct Electrocaloric Measurement

**Figure 9:**
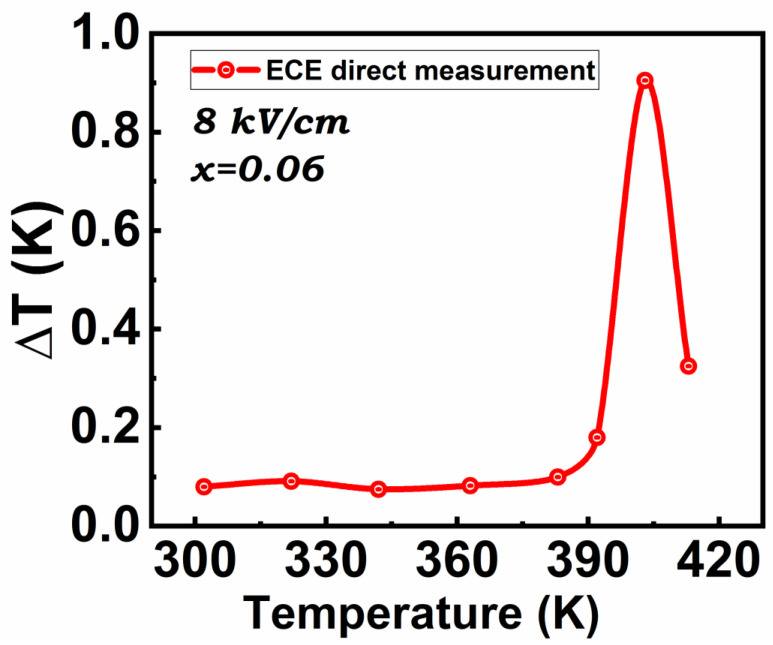
Adiabatic electrocaloric temperature variation obtained from composition BTG6 by the direct method under applied electric field of 8 kV/cm.

Direct electrocaloric measurements have been performed using a modified high-resolution calorimeter on the composition BGT6, which exhibits the highest ECE response in the case of indirect method. As presented in Figure 9, the adiabatic temperature variation in this compound reaches ΔT = 0.9 K under an applied electric field of 8 kV/cm. This value is in an excellent agreement to that obtained by indirect calculation of the adiabatic temperature variation in this compound, whose value was 0.8 K under 7.95 kV/cm. Sharp ECE and dielectric peaks demonstrate the first order character of the ferroelectric transition in BTGx. The latent heat enhancement can explain the relatively large ECE obtained at small field changes at the FE-PE transition, similar to that observed in BTO [6].

Indeed, large ECE responsivity Δ*T/*Δ*E* = 1.13 × 10^−6^ K.m/V was obtained by direct measurements, putting this compound into the category of promising materials for refrigeration applications.

## 6. Energy Storage Investigations

Electrostatic energy storage studies have been investigated. The charged energy density (*W_ch_*), the loss energy density (*W_loss_*), energy storage density (*W_rec_*) and energy storage efficiency (*η*) were calculated from P-E hysteresis data. These physical quantities can be expressed by the following equations, respectively:

(2) $$W_{ch}=\overset{P_{m}}{\underset{0}{\int }}E\;dP,$$

(3) $$W_{rec}=\overset{P_{m}}{\underset{P_{r}}{\int }}E\;dP,$$

(4) $$W_{loss}=W_{ch}-W_{rec},$$

(5) $$\eta =\frac{W_{rec}}{W_{ch}}\times 100,$$

where *P_m_, P_r_* and *E* denote maximum polarization, remnant polarization and electric field strength, respectively. *W_loss_* represents the difference of the energy brought during the charge and that during the discharge process, equivalent to $W_{rec}$ and η is energy storage efficiency coefficient [46,47].

**Figure 10:**
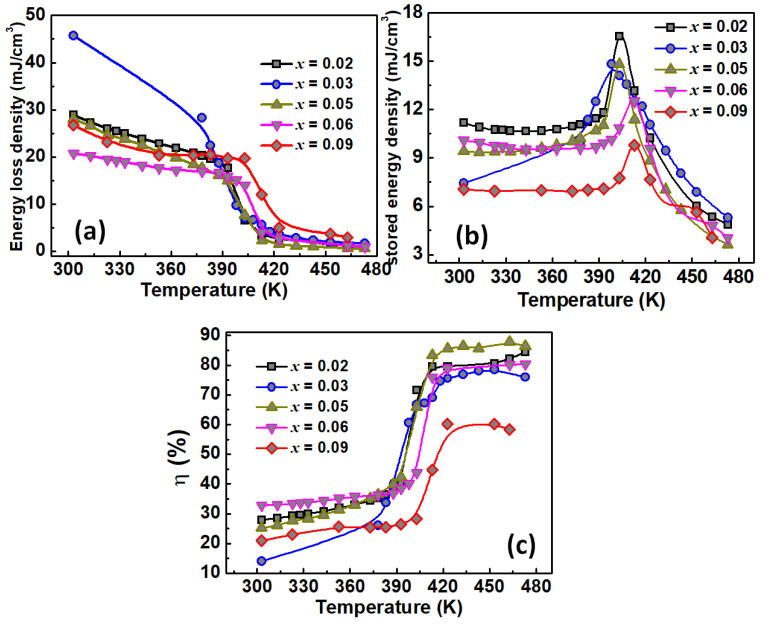
(**a**) Energy lost, (**b**) energy stored and (**c**) energy storage efficiency for all the BTGx compositions (x = 0.02, 0.03, 0.05, 0.06, 0.09). Energy loss decreases globally versus temperature for all the compositions. Maximal energy stored is obtained for BTG2. Higher energy storage efficiency is observed in BTG5.

In Figure 10a–c, we plot energy loss density, energy stored density, and energy storage efficiency coefficient versus temperature for all the studied compounds.

For all the compositions, energy loss density value plotted in Figure 10a decreases and presents a step shape at the Curie point, dropping even more in the paraelectric phase. At the same time, the energy storage density (Figure 10b) shows a λ-shape curve with a maximum around the Curie point, where energy storage appears to be at its maximum. As the energy loss seems to be minimal in the paraelectric phase, this is favourable to a high energy storage efficiency, as shown in Figure 10c in all the studied Ge-content BTGx ceramics.

As expected, the composition BTG6 shows the lower energy lost in accordance with its density and its dielectric and ferroelectric responses. On the other hand, higher energy loss was observed in the ferroelectric phase for the composition BTG3. The sample BTG2, on the other hand, presents a higher energy stored value, and this energy decreases when Ge-content increases. This behaviour is different to the electrocaloric responsivity behaviour, which showed a maximum for BTG6. This result shows the decorrelation between the electrocaloric effect and energy storage mechanism. The former depends on pyroelectric coefficient jump and domain walls dynamic, while the latter depends on the ceramic density and polarization value. Furthermore, as shown in Figure 10b, the higher energy storage density 16.65 mJ/cm^3^ was observed for the composition BTG2 at the Curie temperature. This global small value can be attributed to samples density, the shape of PE-hysteresis loops and maximal polarisation value, since higher values are usually obtained from slimmer PE-hysteresis loops, similarly to relaxor-type ferroelectrics [48,49]. However, the energy storage efficiency of BTGx samples was observed in the range 55 to 88% in the paraelectric phase. The maximum value 87.67% is observed for BTG5. Only the composition BTG9 shows somewhat smaller energy storage efficiency; this Ge concentration approaching limit of solubility. Nevertheless, these results place this family compound in stable energy storable compounds at relatively high temperatures due to the energy storage efficiency.

## 7. Conclusions

High ECE was evidenced in the lead-free BTGx system (*x* = 0.02, 0.03, 0.05, 0.06, 0.09). These compounds exhibit classical ferroelectric behaviour confirmed from P-E hysteresis and FE-PE phase transition confirmed by dielectric and heat capacity measurements. The substitution of titanium (Ti) by germanium (Ge) ions in BTO matrix in the octahedral sites was confirmed by structural analysis based on X-ray diffraction patterns, Raman spectroscopy and SEM images. All compositions are of pure perovskite tetragonal structure and acceptable compactness ceramics. However, Ge-doping did not induce structural symmetry change but the decrease of lattice parameters and volume. Electrical and heat capacity measurements show first-order-type phase transition for all the BTGx compounds, the T_C_ value varies weakly and a thermal hysteresis of about 2.00 K is observed. ECE responsivity was calculated for all the compositions from the indirect method that reveals large values, especially for the compound BTG6 (ΔT/ΔE = 1.01 K.m/V at 400 K), in good agreement with direct EC measurements result of ΔT/ΔE of 1.13 × 10^−6^ K.m/V under 8 kV/cm applied electric field. Energy storage investigations show moderate energy stored of 16.65 mJ/cm^3^ and energy storage efficiency of 87.97%. These results make the lead-free BTGx system a promising alternative candidate for refrigeration and energy storage materials.

## Figures and Tables

**Table 1:** Atomic positions and Rietveld reliability factors for BTGx (x = 0.02, 0.03, 0.05, 0.06, 0.09) compositions.

Atoms	*Atomic* *Positions*	*Compositions*				
		*0.02*	*0.03*	*0.05*	*0.06*	*0.09*
**Ba**	***x***	0	0	0	0	0
	***y***	0	0	0	0	0
	***z***	−0.0422 (4)	0.1827 (5)	−0.0293 (8)	0.3342 (4)	0.1724 (2)
	***Occ.***	1	1	1	1	1
	***B_iso_***	1.664	0.795	0.586	1.074	1.581
**Ti**	***x***	0.5	0.5	0.5	0.5	0.5
	***y***	0.5	0.5	0.5	0.5	0.5
	***z***	0.4798 (5)	0.6677 (3)	0.4874 (5)	0.8681 (3)	0.7117 (2)
	***Occ.***	0.98	0.97	0.95	0.94	0.91
	***B_iso_***	2.1	1.272	1.218	1.433	1.874
**Ge**	***x***	0.5	0.5	0.5	0.5	0.5
	***y***	0.5	0.5	0.5	0.5	0.5
	***z***	0.4798 (5)	0.6677 (3)	0.4874 (5)	0.8681 (3)	0.7117 (2)
	***Occ.***	0.02	0.03	0.05	0.06	0.09
	***B_iso_***	2.1	1.272	1.218	1.433	1.874
**O1**	***x***	0.5	0.5	0.5	0.5	0.5
	***y***	0.5	0.5	0.5	0.5	0.5
	***z***	−0.0481 (3)	0.1413 (8)	−0.0068 (2)	0.2753 (7)	0.0853 (1)
	***Occ.***	1	1	0.9421	1	1
	***B_iso_***	3.331	2.675	1	2.018	3.798
**O2**	***x***	0.5	0.5	0.5	0.5	0.5
	***y***	0	0	0	0	0
	***z***	0.5064 (2)	0.7333 (5)	0.5447 (6)	0.7720 (2)	0.7306 (7)
	***Occ.***	1	1	0.98	1	1
	***B_iso_***	1.974	0.222	2.982	0.438	3.034
***Symmetry Group***		*P*4*mm*	*P*4*mm*	*P*4*mm*	*P*4*mm*	*P*4*mm*
***Unit cell parameters***	***a(Å)***	3.9955 (9)	3.9949 (3)	3.9945 (6)	3.9941 (7)	3.9905 (5)
	***c(Å)***	4.0364 (6)	4.0350 (5)	4.0358 (3)	4.0324 (5)	4.0309 (5)
***Unit cell Volume V (Å^3^)***		64.4371 (7)	64.3954 (8)	64.3953 (5)	64.3282 (1)	64.1884 (2)
***Nb Refined param.***		45	33	37	38	34
***Grain size (µm)***		3.63	5.45	10.90	8.32	18.77
***Theoretical Density (g/cm^3^)***		6.067	6.052	6.045	6.041	6.049
***Calculated Density (g/cm^3^)***		5.801	5.785	5.645	5.035	5.648
***Relative Density (%)***		96	95	93	83	93
***χ^2^***		2.85	2.1	2.14	2.5	3.72
***Rwp***		5.57	3.84	3.07	3.18	4.62
***R_P_***		4.23	2.71	2.21	2.14	3.19
***R_Bragg_***		4.64	6.54	5.26	3.37	3.5
***Ti-O_1_ (c-axis)***		1.8278 (7)	1.7198 (8)	1.9947 (7)	2.0302 (3)	1.8998 (6)
***Ti-O_2_ (c-axis)***		2.2086 (6)	2.3151 (0)	2.0410 (2)	2.0023 (4)	2.1311 (3)
***Goldschmidt factor***		0.9929	0.9932	0.9940	0.9944	0.9956
***Curie C (×10^5^ K)***		3.78	3.42	3.87	3.40	3.44
***T_0_ (K) ± 1.00 K***		372.11	353.79	359.13	348.63	354.40
***T_C_ (K) ± 0.12 K***		400.83	399.40	400.10	399.73	399.53

## Data Availability

Not applicable.
